# Diagnostic accuracy of complete blood cell count and neutrophil-to-lymphocyte, lymphocyte-to-monocyte, and platelet-to-lymphocyte ratios for neonatal infection

**DOI:** 10.2478/abm-2022-0006

**Published:** 2022-02-28

**Authors:** Abdullah Kurt, Merve Sezen Tosun, Nilgün Altuntaş

**Affiliations:** Department of Pediatrics, Ankara Yildirim Beyazit University Yenimahalle Training and Research Hospital, Ankara 06370, Turkey

**Keywords:** infant, newborn, infections, lymphocytes, monocytes, neutrophils, sepsis

## Abstract

**Background:**

Complete blood cell (CBC) counts and neutrophil-to-lymphocyte (NLR), lymphocyte-to-monocyte (LMR), and platelet-to-lymphocyte ratios (PLR) are simple measurements that are conducted as part of routine diagnostic procedures.

**Objective:**

To determine the diagnostic importance, specificity, and sensitivity of these measurements for the diagnosis of neonatal infections and in discriminating between neonatal sepsis and various other infections.

**Methods:**

We conducted a retrospective study of data from a consecutive series of 232 neonatal patients admitted to Yildirim Beyazit University Yenimahalle Training and Research Hospital in Ankara for 2 years from 2016 to 2018. We included patients with a diagnosis of or clinically suspected infection, and healthy neonates were included as controls. Data included CBC counts, and bacterial culture results, considered the criterion standard for the diagnosis of neonatal sepsis. NLR, LMR, and PLR were calculated. We compared data using independent Student *t* and Mann–Whitney *U* tests and determined the sensitivity, specificity, and likelihood ratio (LHOR) of the characteristics for neonatal sepsis using receiver operating characteristic curve analyses.

**Results:**

We included data from 155 neonatal patients with a diagnosis or suspicion of infection and 77 healthy neonates. NLR was significantly higher in neonates with sepsis or fever due to dehydration (*P* < 0.001) than in neonates with other infections or healthy neonates. LMR was significantly higher in neonates with sepsis or viral infection than in those with other infections or healthy controls (*P* = 0.003). In neonates with early-onset sepsis (EOS), we found cut-off values of ≥4.79 [area under curve (AUC) 0.845, 95% confidence interval (CI) 0.76–0.93, LHOR 11.6, specificity 98.7%, sensitivity 15%] for NLR, ≥1.24 (AUC 0.295; CI 0.18–0.41, LHOR 1.02, specificity 2.6%, sensitivity 100%) for LMR, and ≥37.72 (AUC 0.268; CI 0.15–0.39, LHOR 0.86, specificity 7.8%, sensitivity 80%) for PLR. We found cut-off values of ≥4.94 (AUC 0.667; CI 0.56–0.77, LHOR 4.16, specificity 98.7%, sensitivity 5.4%) for NLR and ≥10.92 (AUC 0.384; CI 0.26–0.51, LHOR 6.24, specificity 98.7%, sensitivity 8.1%) for LMR in those with late-onset sepsis (LOS).

**Conclusions:**

CBCs, NLR, LMR, and PLR may be useful for the differential diagnosis of EOS and LOS, and neonates with sepsis from those with other infection. NLR may be a useful diagnostic test to identify neonatal patients with septicemia more quickly than other commonly used diagnostic tests such as blood cultures. NLR has high specificity and LHOR, but low sensitivity.

Sepsis is a common cause of mortality and morbidity in newborns in their first month of life (neonates) [1, 2]. Neonatal sepsis is considered a systemic condition of bacterial infection that is associated with hemodynamic changes and other clinical manifestations and laboratory findings. Although blood culture is the criterion standard for the diagnosis of neonatal sepsis, it is a disadvantage for early diagnosis that the culture results cannot be available before 24–48 h and that false negative and positive results can be obtained. Various laboratory biomarkers such as interleukins (ILs), tumor necrosis factor (TNF), C-reactive protein (CRP), procalcitonin (PCT), and immunoglobulins have been considered for the diagnosis of neonatal sepsis [3, 4]. An important association for infection risk in neonates is a qualitative and quantitative deficiency of neutrophils. Neutropenia associated with neonatal sepsis adversely affects prognosis [2, 5,6,7,8,9]. To our knowledge, Záhorec et al. [10] first recommended use of the neutrophil-to-lymphocyte ratio (NLR) as a marker of infection [4]. A study of adults by Loonen et al. [11] found that NLR was a diagnostic characteristic of patients with culture-positive sepsis compared with patients without culture-positive sepsis (culture-negative or clinical sepsis). They reported a NLR of 23.0 ± 15.0 [mean ± standard deviation (SD)] in patients with positive blood cultures and 12.2 ± 9.1 in the group with negative cultures [*P* < 0.001; area under the curve (AUC) for NLR 0.77; 95% confidence interval (CI) 0.66–0.88] and considered NLR promising for a rapid assessment [11]. Also in adults, de Jager et al. [12] found significant differences in the NLR between patients with positive and negative blood cultures (20.9 ± 13.3 vs. 13.2 ± 14.1; *P* < 0.0001). Furthermore, sensitivity was 77.2%, specificity 63.0%, and positive and negative predictive values (67.6% and 73.4%, respectively) were highest for the NLR with an AUC of 0.73 (95% CI: 0.66–0.81). They suggested that NLR is a better predictor of bacteremia than CRP, white blood cell count (WBC), or neutrophil count [12]. In more recent studies, complete blood cell (CBC) counts have been used in the diagnosis and prognosis of various diseases [13, 14].

Physiological changes occur in the peripheral blood cells in newborns, in the first few days of life [15, 16]. Normally, the neutrophil count increases in this period and later decreases [15, 16]. The changes in neutrophil count may be associated with the normal physiological response or a disorder (such as infection). The aim of the present study was to determine the diagnostic importance, specificity, and sensitivity of peripheral blood cell counts, NLR, lymphocyte-to-monocyte ratio (LMR), and platelet-to-lymphocyte ratio (PLR) in neonatal infections and in discriminating between neonatal sepsis and various other infections.

## Methods

### Study design

#### Patients

We conducted a retrospective study of data from a consecutive series of patients who were admitted to the neonatology outpatient clinic and neonatal intensive care unit (NICU) of the Yildirim Beyazit University Yenimahalle Training and Research Hospital in Ankara for 2 years from 2016 to 2018 (total enumerative sampling). This study was approved by the Yildirim Beyazit University Yenimahalle Training and Research Hospital Clinical Research Ethics Committee (approval No. 2018/0508) and was conducted in accordance with Turkish national guidelines and the principles of the contemporary revision of the Declaration of Helsinki (64th WMA General Assembly 2013). Documented informed consent was specifically waived by the committee because the study was retrospective. Patient data were obtained from the hospital electronic record system and included postnatal age, sex, birthweight, gestational week, hospital stay, peripheral CBC, WBC, lymphocyte, monocyte, neutrophil, platelet count, CRP, and culture (blood, urine) results during the hospitalization. NLR, LMR, and PLR were calculated. The study included neonatal patients with a diagnosis or suspicion of infection, and healthy neonatal patients as controls. The control group included a consecutive series of healthy full-term newborns who had no infections or any referral to the NICU according to their history, and whose physical examination and laboratory test results were accepted to be within the normal/healthy reference range.

We excluded data from patients for whom file information was inaccessible or incomplete, and from patients with genetic disease, metabolic disease, congenital heart disease, or perinatal asphyxia.

#### Diagnoses

Data from all newborns suspected with infection during the 2-year admission period were included in the study (total enumerative sampling). Data from consecutive patients hospitalized in our NICU who met inclusion criteria were included. The neonatal patient group was divided into 6 subgroups according to their final diagnosis, which was supported by clinical examination, microbiology, serology, and radiology. The 6 subgroups were neonatal sepsis (including early-onset neonatal sepsis and late-onset neonatal sepsis), pneumonia, urinary tract infection (UTI), focal infection, and viral infection. Neonatal sepsis was defined as a clinical syndrome in an infant within 28 d of life or younger, manifested by systemic signs of bacterial infection, and/or isolation of a bacterial pathogen from the bloodstream.

Blood samples were taken from all newborns immediately after hospitalization and used for CBCs and blood cultures. Patients with neonatal sepsis comprised patients with positive blood culture, or with negative blood culture, but clinically recognized sepsis. Patients with neonatal sepsis were categorized as those with early-onset sepsis (EOS) and late-onset sepsis (LOS). EOS was defined as the onset of symptoms before 4 days old. LOS was defined as the onset of symptoms at ≥4 days old. comprised Patients without signs of sepsis or systemic infection or positive blood culture comprised other neonatal patients with infection. We followed the STARD reporting guidelines for studies of diagnostic accuracy [17].

### Statistical analysis

Data were analyzed using IBM SPSS Statistics for Windows (version 19). A Kolmogorov–Smirnov test was used to determine normal distribution of data. Numerical data were compared with the control group data using independent Student *t* or Mann–Whitney *U* tests. Categorical data were compared using a χ^2^ test. Sensitivity, specificity, and likelihood ratio (LHOR) were determined using a receiver operating characteristic (ROC) curve analysis. In the ROC curve, there are points corresponding to various combinations of sensitivity and specificity values. Because both the sensitivity and the specificity of the test should be high, the selected cut-off value should be the lowest 1 – specificity point versus the highest sensitivity value. One way to find this point is to calculate the LHOR. The cut-off limit of the maximum value for LHOR was found from the obtained coordinate table, and the calculation was made from the “LHOR = sensitivity/(1–specificity)” formula. *P* < 0.05 was considered significant.

## Results

Data from 232 neonates aged 1–28 d were included in the study. Data were allocated into 2 groups: a neonatal patient group including 155 newborns with infection or fever in the NICU and a control group including 77 healthy newborn patients. The 232 patients included in this study of a 2-year admission period included 0.7% of the total number (about 30,100 newborns) admitted to the neonatology outpatient clinic of our hospital. The present study includes data from 57 patients with neonatal sepsis, 44 patients with pneumonia, 18 patients with UTI, 12 patients with focal infection, and 9 patients with viral infection.

### Neonatal sepsis

Culture-positive sepsis (proven sepsis) was identified in 22 neonatal patients whose clinical and laboratory findings were consistent with sepsis, and the causative agent was demonstrated. Clinical sepsis was identified in another 35 neonatal patients whose clinical and laboratory findings were consistent with sepsis, but an agent was not or could not be demonstrated.

### Pneumonia

Pneumonia was diagnosed according to clinical, laboratory, and radiological findings in 44 neonatal patients.

### Urinary tract infections

UTI were diagnosed according to urine culture and clinical or laboratory findings in 18 neonatal patients.

### Focal infections

According to clinical and laboratory findings, 51 neonatal patients had omphalitis, pyoderma, cellulitis, conjunctivitis, and other focal infection.

### Viral infections

Patients having viral infections were admitted to the NICU with mild respiratory distress, runny nose, mild fever, and poor feeding. Radiological examinations, CRP values, and culture results were determined to be within the age-appropriate reference range. In these patients, pneumonia, bacterial infection, and sepsis were excluded, and only supportive treatment was given. This group comprised 9 patients diagnosed with upper respiratory tract infection due to clinical findings. However, in our hospital conditions, viral tests could not be performed to ascertain the possible agent of viral infections in these patients.

### Fever (no infection)

Newborns who had only fever and >10% weight loss during their referral were hospitalized for suspicion of sepsis. This group comprised 15 patients who did not have growth of any organism in any culture and were without clinical and laboratory findings of sepsis.

### Control group

**Figure 1:**
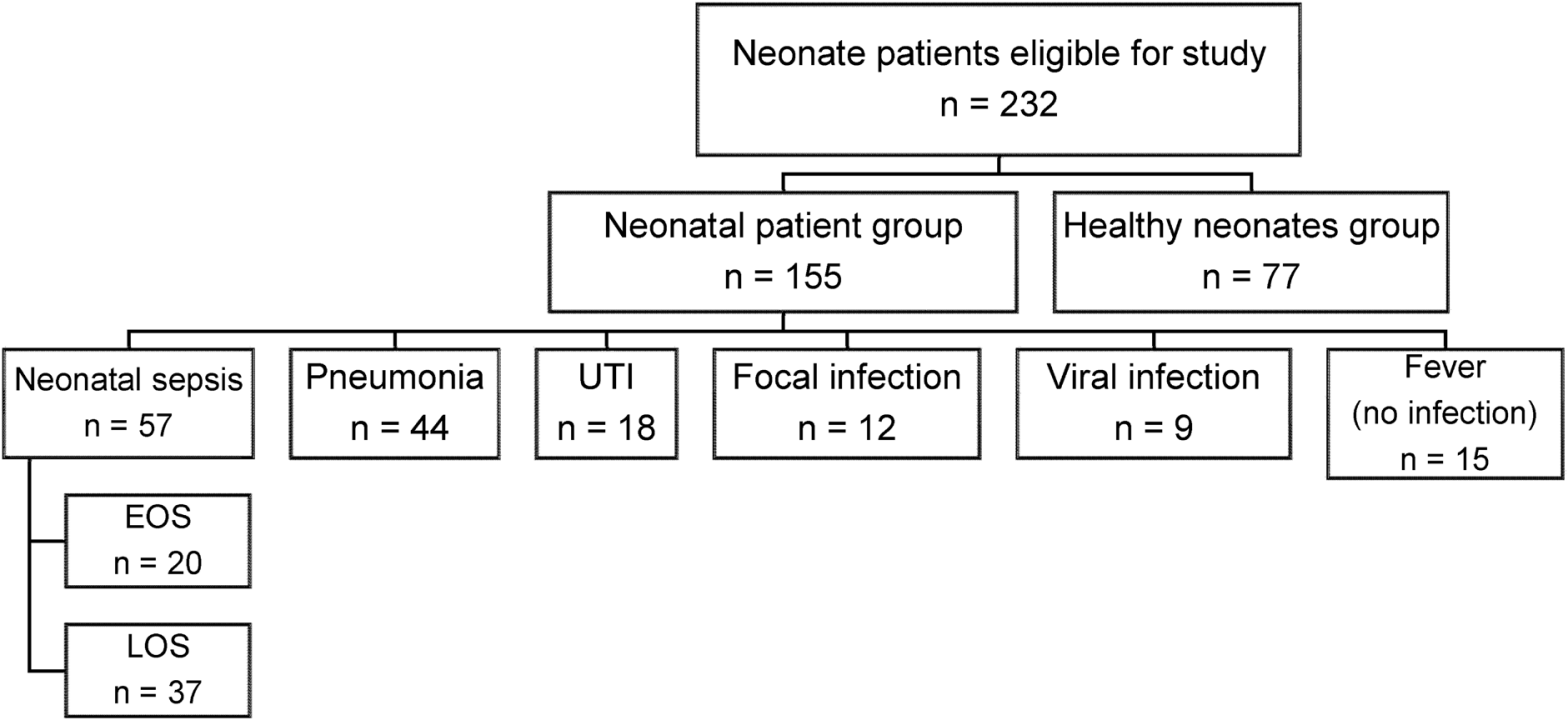
Diagram of categories of neonatal patients whose data were included.

A total of 77 healthy newborns who were admitted the neonatology outpatient clinic were included in the control group (**Figure 1**).

There was no significant difference in demographic characteristics between the neonatal patient group and the control group (*P* > 0.05, **Table 1**).

**Table 1:** Patient demographic characteristics

**Characteristic**	**Neonatal patient group (n = 155)**	**Healthy neonates (n = 77)**	***P***
Sex, n (%)	Female 65 (41.9)	Female 34 (44.2)	0.77†
	Male 90 (58.1)	Male 43 (55.8)	
Form of birth, n (%)	Normal vaginal route 97 (62.6)	Normal vaginal route 41 (53.2)	0.20†
	Cesarean section 58 (37.4)	Cesarean section 36 (46.8)	
Gestational weeks¶	38.8 ± 1.13 (37–42)	38.7 ± 1.06 (37–41)	0.63‡
Birth weight (g)¶	3,397 ± 468 (1,830–4,850)	3,362 ± 372 (2,400–4,050)	0.57§
Postnatal age (d)¶	14.4 ± 9.6 (1–28)	12.2 ± 8.5 (1–28)	0.14‡

¶ Data are presented as mean ± SD (range: minimum–maximum).

SD, standard deviation.

† χ^2^ test.

‡ Mann–Whitney *U* test.

§ Independent *t* test.

WBC (*P* < 0.001), neutrophil (*P* < 0.001), and monocyte (*P* = 0.016) counts were significantly higher in the neonatal patients with sepsis than in healthy neonatal controls (**Table 2**). NLR was significantly higher in neonatal patients with sepsis (*P* < 0.001) or fever due to dehydration (*P* = 0.001) than it was in those with other infection or healthy neonatal controls. LMR was significantly lower in neonatal patients with sepsis (*P* = 0.004) and those with viral infection (*P* = 0.01) than is was in those with other conditions or healthy neonatal controls.

**Table 2:** Comparison of NLR, LMR, PLR, and peripheral complete blood count parameters between the neonatal patient group and healthy neonatal control group

	**Neonatal patient group (n** = **155) Median** ± **IQR (percentiles: 25th–75th)**						

	**Neonatal sepsis (n = 57)**	**Pneumonia (n = 44)**	**UTI (n = 18)**	**Focal infection (n = 12)**	**Viral infection (n = 9)**	**Fever (no infection) (n = 15)**	**Healthy neonatal control group (n = 77)** **Median ± IQR (percentiles 25th–75th)**
WBC (×10^9^/L)	14.2 ± 8.82 (10.22–19.03)^*^	11.10 ± 5.59 (8.48–13.57)	11.57 ± 5.53 (8.87–14.40)	13.85 ± 11.8 (9.24–21.05)	10.2 ± 4.10 (8.75–12.85)	13.90 ± 6.80 (10.9–17.7)^*^	10.12 ± 4.84 (8.36–13.20)
Lymphocytes (×10^9^/L)	3.97 ± 2.58 (2.92–5.50)	4.98 ± 2.76 (3.46–6.22)	4.66 ± 2.68 (3.52–6.20)	5.26 ± 3.58 (3.57–7.15)	4.56 ± 2.30 (2.92–5.21)	3.73 ± 1.30 (3.54–4.84)	4.53 ± 1.52 (3.87–5.39)
Neutrophils (×10^9^/L)	7.61 ± 8.35 (3.85–12.20)^*^	3.33 ± 3.63 (2.32–5.95)	4.46 ± 4.20 (2.37–6.58)	4.0 ± 9.24 (2.13–11.37)	3.94 ± 2.26 (3.01–5.26)	8.41 ± 5.90 (4.60–10.5)^*^	3.38 ± 3.13 (2.29–5.42)
Monocytes (×10^9^/L)	1.41 ± 1.06 (0.83–1.89)^*^	1.28 ± 1.21 (0.82–2.03)^*^	1.39 ± 0.94 (0.83–1.77)	1.75 ± 1.17 (1.13–2.30)^*^	1.64 ± 1.77 (1.15–2.92)^*^	1.05 ± 0.94 (0.81–1.75)	1.21 ± 0.67 (1.21–1.49)
Platelets (×10^9^/L)	297.0 ± 171.0 (205.5–376.5)	373.5 ± 151.5 (298.25–449.75)	356.0 ± 223.5 (221.0–444.5)	326.0 ± 179.75 (236.25–416.0)	368.0 ± 137.5 (274.5–412.0)	320.0 ± 152.0 (273.0–425.0)	321.0 ± 162.0 (282.5–444.5)
NLR	1.95 ± 2.12 (0.90–3.03)^*^	0.69 ± 0.93 (0.46–1.39)	0.97 ± 0.87 (0.48–1.35)	1.12 ± 1.34 (0.33–1.67)	1.02 ± 1.32 (0.57–1.89)	1.80 ± 1.75 (1.13–2.88)^*^	0.81 ± 0.87 (0.49–1.36)
LMR	2.79 ± 2.03 (2.32–4.35)^*^	3.31 ± 3.42 (2.63–6.05)	4.28 ± 4.02 (2.37–6.38)	2.65 ± 3.30 (2.17–5.46)	1.60 ± 3.71 (1.36–5.08)^*^	3.52 ± 3.32 (2.72–6.05)	3.97 ± 3.09 (2.95–6.05)
PLR	69.25 ± 48.96 (50.66–99.63)	72.6 ± 52.29 (54.74–107.02)	62.78 ± 58.90 (49.99–108.90)	59.77 ± 33.91 (48.10–82.01)	77.31 ± 66.21 (70.6–136.82)	78.40 ± 50.73 (62.14–112.87)	77.90 ± 38.76 (60.40–99.16)
CRP (< 0.5 mg per 100 mL, normal)	0.58 ± 1.69 (0.10–1.79)	0.10 ± 0.54 (0.03–0.56)	0.09 ± 1.10 (0.02–1.12)	0.45 ± 13.64 (0.05–13.69)	0.33 ± 0.59 (0.09–0.68)	1.5 ± 3.11 (0.74–3.85)	–
Hospital stay (d)	9.0 ± 3.0 (–)	9.0 ± 3.0 (–)	10.0 ± 4.0 (–)	10.0 ± 4.75 (–)	6.0 ± 3.0 (–)	8.0 ± 3.0 (–)	–

CRP, C-reactive protein; EOS, early-onset sepsis; IQR, interquartile range; LMR, lymphocyte/monocyte ratio; LOS, late-onset sepsis; NLR, neutrophil/lymphocyte ratio; PLR, platelet/lymphocyte ratio; UTI, urinary tract infection; WBC, white blood cell.

* *P* < 0.05 from independent *t* test or Mann–Whitney *U* test compared with healthy neonates (control group).

WBC (*P* < 0.001), neutrophil (*P* < 0.001), monocyte (*P* = 0.002), platelet counts (*P* < 0.001), and NLR (*P* < 0.001), were significantly higher, and LMR (*P* = 0.005) and PLR (*P* = 0.001) were significantly lower in neonates with EOS than those in healthy controls. Neutrophil counts (*P* = 0.009) and NLR (*P* = 0.004) were significantly higher and LMR (*P* = 0.045) was significantly lower in neonates with LOS than those in healthy controls (**Table 3**).

**Table 3:** Comparison of NLR, LMR, PLR, and peripheral complete blood count parameters between the neonatal sepsis group and healthy neonates control group

	**Neonatal sepsis group (n = 57)** **Median ± IQR (percentiles: 25^th^–75^th^)**		**Healthy neonate group (n = 77)** **Median ± IQR (percentiles: 25^th^–75^th^)**	***P* †**	***P* ‡**
		
	**EOS (n = 20)**	**LOS (n = 37)**			
WBC (×10^9^/L)	18.8 ± 11.27 (13.75–25.02)^*^	13.1 ± 6.57 (8.98–15.56)	10.12 ± 4.84 (8.36–13.20)	<0.001^*^	0.51
Lymphocytes (×10^9^/L)	4.79 ± 1.81 (3.81–5.62)	3.84 ± 2.75 (2.75–5.49)	4.53 ± 1.52 (3.87–5.39)	0.614	0.57
Neutrophils (×10^9^/L)	11.87 ± 7.98 (7.46–15.45)^*^	5.41 ± 7.28 (3.05–10.33)^*^	3.38 ± 3.13 (2.29–5.42)	<0.001^*^	0.009#
Monocytes (×10^9^/L)	1.68 ± 0.99 (1.16–2.15)^*^	1.35 ± 0.86 (0.74–1.59)	1.21 ± 0.67 (1.21–1.49)	0.002^*^	0.36
Platelets (×10^9^/L)	252.5 ± 113.0 (191.5–304.5)^*^	338.0 ± 157.5 (241.0–398.5)	321.0 ± 162.0 (282.5–444.5)	<0.001^*^	0.52
NLR	2.39 ± 1.76 (1.57–3.33)^*^	1.49 ± 2.18 (0.68–2.86)^*^	0.81 ± 0.87 (0.49–1.36)	<0.001^*^	0.004#
LMR	2.74 ± 1.64 (2.41–4.05)^*^	2.84 ± 3.33 (2.21–5.54)^*^	3.97 ± 3.09 (2.95–6.05)	0.005^*^	0.045#
PLR	55.50 ± 34.87 (39.92–74.78)^*^	81.29 ± 51.27 (63.59–114.86)	77.90 ± 38.76 (60.40–99.16)	0.001^*^	0.48

EOS, early-onset sepsis; IQR, interquartile range; LMR, lymphocyte/monocyte ratio; LOS, late-onset sepsis; NLR, neutrophil/lymphocyte ratio; PLR, platelet/lymphocyte ratio; WBC, white blood cell.

* *P* < 0.05;

† Mann–Whitney *U* test compared with the healthy neonates (control group) for EOS.

# *P* < 0.05;

‡ Mann–Whitney *U* test compared with the healthy neonates (control group) for LOS.

**Figure 2:**
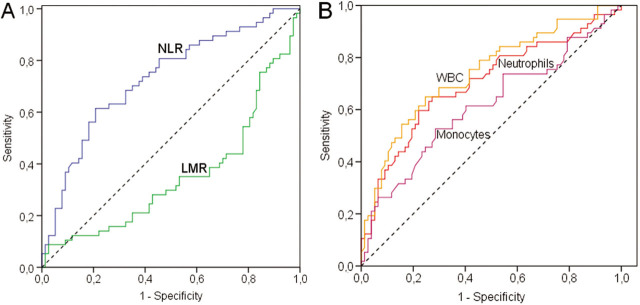
ROC curve analysis for (A) NLR (blue line), LMR (green line), and reference (dashed line); and (B) WBC (orange line), neutrophils (red line), monocytes (mauve), and reference (dashed line) in neonatal sepsis. LMR, lymphocyte/monocyte ratio; NLR, neutrophil/lymphocyte ratio; ROC, receiver operating characteristic; WBC, white blood cell.

**Figure 3:**
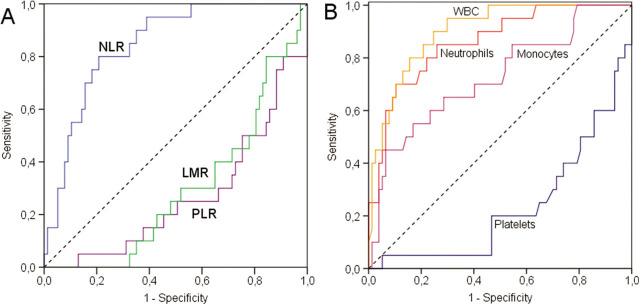
ROC curve analysis for (A) NLR (blue line), LMR (green line), PLR (purple line), and reference (dashed line); and (B) WBC (orange line), neutrophils (red line), monocytes (mauve line), platelets (dark blue line), and reference (dashed line) in EOS. EOS, early-onset sepsis; LMR, lymphocyte/monocyte ratio; NLR, neutrophil/lymphocyte ratio; ROC, receiver operating characteristic; WBC, white blood cell.

**Figure 4:**
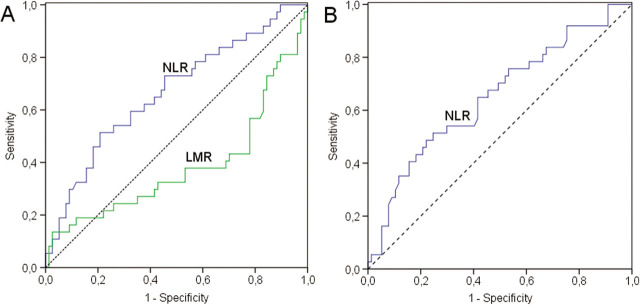
ROC curve analysis for (A) NLR (blue line), LMR (green line), and reference (dashed line); and (B) NLR (blue line), and reference (dashed line) in LOS. LOS, late-onset sepsis; LMR, lymphocyte/monocyte ratio; NLR, neutrophil/lymphocyte ratio; ROC, receiver operating characteristic.

We performed ROC curve (**Figures 2–4**) analysis for NLR and LMR with WBC, neutrophil, and monocytes in neonatal patients with sepsis, and sensitivity, specificity, and LHOR are presented in **Table 4**. High specificity and low sensitivity were found for CBCs to exclude sepsis in neonatal patients suspected of having neonatal sepsis. These associations are stronger for EOS than LOS. LHOR for WBC was 10.2, neutrophil count was 30.8, and NLR was 11.5, with high specificity but low sensitivity. Platelet counts were not found effective in the diagnosis of neonatal sepsis.

**Table 4:** ROC analysis results for NLR, LMR, PLR, and peripheral blood count in patients with neonatal sepsis

**Variable**	**Sensitivity (%)**	**Specificity (%)**	**LHOR**	**AUC ROC**	**95% Confidence interval**	**Cut-off**	***P***
Neonatal sepsis							
WBC (×10^9^/L)	10.5	98.7	8.1	0.700	0.61–0.79	≥ 23.4	<0.001^*^
Neutrophils (×10^9^/L)	15.8	98.7	12.1	0.739	0.65–0.83	≥14.2	<0.001^*^
Monocytes (×10^9^/L)	21.1	96.1	5.4	0. 613	0.51–0.71	≥2.04	0.025^*^
NLR	8.8	98.7	6.7	0.729	0.64–0.82	≥4.79	<0.001^*^
LMR	5.3	98.7	4.05	0.353	0.26–0.45	≥10.9	0.004^*^
EOS							
WBC (×10^9^/L)	40	96.1	10.26	0.861	0.77–0.95	≥20.4	<0.001^*^
Neutrophils (×10^9^/L)	40	98.7	30.8	0.902	0.84–0.97	≥14.0	<0.001^*^
Monocytes (×10^9^/L)	10	98.7	7.7	0.725	0.59–0.86	≥2.77	0.002^*^
Platelets (×10^9^/L)	5	94.8	0.96	0.236	0.12–0.35	≥526	<0.001^*^
NLR	15	98.7	11.6	0.845	0.76–0.93	≥4.79	<0.001^*^
LMR	100	2.6	1.02	0.295	0.18–0.41	≥1.24	0.005^*^
PLR	80	7.8	0.86	0.268	0.15–0.39	≥37.7	0.001^*^
LOS							
Neutrophils (×10^9^/L)	5.4	98.7	4.16	0.651	0.54–0.76	≥14.5	0.009^*^
NLR	5.4	98.7	4.16	0.667	0.56–0.77	≥4.94	0.004^*^
LMR	8.1	98.7	6.24	0.384	0.26–0.51	≥10.9	0.045^*^

AUC, area under curve; EOS, early-onset sepsis; LHOR, likelihood ratio; LMR, lymphocyte/monocyte ratio; LOS, late-onset sepsis; NLR, neutrophil/lymphocyte ratio; PLR, platelet/lymphocyte ratio; ROC, receiver operating characteristic; WBC, white blood cell.

* *P* < 0.05 from independent *t* test or Mann–Whitney *U* test compared with healthy neonates (control group).

## Discussion

CBC contributes to differential diagnosis of neonatal sepsis in newborns [1, 2, 18,19,20]. WBC, absolute neutrophil counts (ANCs), and the ratio of immature to total neutrophils (I:T) may be used in the diagnosis of neonatal sepsis. For diagnosis of neonatal sepsis, low WBC values (<5,000 mm^3^) are more important. Although WBC, ANC, and I:T ratio are commonly used in the diagnosis of neonatal sepsis, these parameters have limitations. Christensen et al. [20] reported that due to their low sensitivity these CBC-derived indices are not reliable diagnostic markers to exclude EOS in neonates because there is no change in CBC in the first hours of sepsis in neonates with EOS and concluded CBC-derived indices are a poor indicator. These tests have been confirmed by others as not specific for the diagnosis of sepsis [2, 8, 21,22,23,24]. By contrast, there are studies that did find that peripheral blood count results are useful in the diagnosis of sepsis [9, 18]. Murphy et al. [18] showed that sterile blood culture and 2 normal I:T ratios were 100% negative predictive values in the diagnosis of the EOS. Philip et al. [9] showed that neutropenia was better predictive of neonatal sepsis than neutrophilia.

WBC may be normal or very mildly low or accompanied by leukopenia in neonatal infections with enterovirus, herpes simplex virus 1 and 2 (HSV), and human parechoviruses (HPeV) [25]. We found that WBC (*P* = 0.003) and neutrophil counts (*P* = 0.001) were significantly higher in neonates who presented with fever without infection than those in healthy neonatal controls. Monocyte counts of the neonatal patients were significantly higher than the counts in the healthy neonatal controls, specifically in patients with neonatal sepsis (*P* = 0.016), pneumonia (*P* = 0.025), focal infection (*P* = 0.009), and viral infection (*P* = 0.014). Monocytes counts were higher in neonatal patients with viral infections than they were in those with other infections. (**Table 2**).

Manzoni et al. [26] reported that thrombocytopenia is not an organism-specific marker of sepsis. Low platelet counts should not be associated with any infectious agent (or agent group) in preterm newborns. Platelet counts can be used as a prediagnostic test for neonatal sepsis, but are not very specific to neonatal sepsis. However, platelet counts can help to monitor treatment prognosis [1, 2, 25,26,27].

Peripheral CBCs are used for diagnosis in pediatric patients with various ages and diseases [28,29,30]. Naess et al. [28] reported that NLR is a more useful diagnostic tool than other blood tests that are used to identify patients with septicemia. They reported that NLR and MLR may be useful in the differential diagnosis of bacterial infection among patients hospitalized for fever [28]. Warimwe et al. [29] reported that MLR, which is easily derived from routine peripheral CBC counts in children diagnosed with malaria, is an effective method for demonstrating their immune status against *Plasmodium falciparum* infection. Mentis et al. [30] reported that cerebrospinal fluid NLR can serve as an additional biomarker for the differential diagnosis between bacterial and viral meningitis.

We determined the cut-off for NLR, LMR, and PLR for EOS and LOS (**Table 4**). Russell et al. [31] evaluated the utility of NLR, LMR, and PLR as infection biomarkers in their meta-analysis of 10 studies that found a relationship between NLR and bacteremia, with a cut-off >12.65 for NLR (n = 3,320 AUC 0.72, *P* < 0.0001). Two studies found an association between low LMR and the diagnosis of influenza virus infection in patients with respiratory tract infection, with a cut-off ≤2.06 for LMR (n = 85; AUC 0.66, *P* = 0.01). A study in adults found potential utility for NLR in pneumonia, pertussis, UTI, and diabetic foot infections, and PLR in Crimean–Congo hemorrhagic fever [31]. LMR may be useful in diagnosis of respiratory virus infection (especially in influenza virus infections) in adults, and NLR is useful for estimating mortality during sepsis and bacteremia [31]. In the present study of neonatal patients with sepsis, we found a cut-off ≥4.79 for NLR and ≥10.9 for LMR, and in neonates with EOS a cut-off ≥4.79 for NLR, ≥1.24 for LMR, and ≥37.72 for PLR. In neonates with LOS, we found a cut-off ≥4.94 for NLR and ≥10.92 for LMR (**Table 4**). There appears a lack of data in the literature for the diagnostic accuracy of NLR, LMR, and PLR in neonatal sepsis. To our knowledge, the present study is the first to determine the diagnostic accuracy of NLR, LMR, and PLR in neonatal sepsis.

We consider that CBC counts can contribute to the diagnosis of neonatal sepsis according to various groups. NLR and WBC, neutrophil, and monocyte counts had high specificity in neonates with EOS. These measures could be used to distinguish EOS from other diseases and guide the empirical use of antibiotics. In the neonatal patients with fever without sepsis, NLR, WBC, and neutrophil counts were also significantly higher than those in healthy neonates in the control group. However, these patients were dehydrated because of poor feeding. We consider that, in these patients, increases of the WBC and neutrophil counts and NLR may be related to dehydration because of the relative neutrophilia.

A limitation of the present study is that while newborns with infectious diseases were included, those with other disorders were excluded. Considering the physiological changes in the peripheral blood count in the first few days of newborns, it can be difficult to determine the association of blood counts with infection. Not all neonatal patients considered with sepsis here had a positive blood culture result. The subsample sizes are relatively small in this single-center study from Turkey and results may not be generalizable to other populations. Larger multicenter studies with a longer sample time to include larger sample sizes are warranted.

## Conclusions

Knowing the number of WBCs, neutrophils, and monocytes may contribute to the diagnosis of neonatal sepsis. CBCs, NLR, LMR, and PLR may be useful for the differential diagnosis of EOS and LOS, and neonates with sepsis and other infection. CBCs and NLR have high specificity and LHOR, but low sensitivity. NLR may be a useful diagnostic test with which to identify neonatal patients with sepsis more quickly than other commonly used diagnostic tests, such as blood cultures, guiding the early empirical use of antibiotics.
